# Supplementary material for: In situ evidence of the magnetospheric cusp of Jupiter from Juno spacecraft measurements
Source: Nat Commun. 2024 Jul 18;15:6062. doi: 10.1038/s41467-024-50449-z (PMC11258361; doi:10.1038/s41467-024-50449-z)
Supplement: Supplementary file 1 — Supplementary Information [file 41467_2024_50449_MOESM1_ESM.pdf]

In Situ Evidence of the Magnetospheric Cusp of Jupiter from Juno Spacecraft Measurements

Y. Xu[1,2,3], C. S. Arridge[3], Z. H. Yao[1,2,4,5]*, B. Zhang[4], L. C. Ray[3], S. V. Badman[3], W. R. Dunn[5], R. W. Ebert[6,7], J. J. Chen[4], F. Allegrini[6,7], W. S. Kurth[8], T. S. Qin[4], J. E. P. Connerney[9,10], D. J. McComas[11], S. J. Bolton[6], Y. Wei[1,2]

[1] Key Laboratory of Earth and Planetary Physics, Institute of Geology and Geophysics, Chinese Academy of Sciences, Beijing, China

[2] College of Earth and Planetary Sciences, University of Chinese Academy of Sciences, Beijing, China

[3] Department of Physics, Lancaster University, Lancaster, UK

[4] NWU-HKU Joint Centre of Earth and Planetary Sciences, Department of Earth Sciences, University of Hong Kong, Hong Kong SAR, China

[5] Department of Physics and Astronomy, University College London, London, UK

[6] Southwest Research Institute, San Antonio, TX, USA

[7] Department of Physics and Astronomy, University of Texas at San Antonio, San Antonio, TX, US

[8] Department of Physics and Astronomy, University of Iowa, Iowa City, IA, USA

[9] Space Research Corporation, Annapolis, MD, USA

[10] NASA/Goddard Space Flight Center, Greenbelt, MD, USA,

[11] Department of Astrophysical Sciences, Princeton University, Princeton, NJ, USA

Correspondence and requests for materials should be addressed to Zhonghua Yao (yaozh@hku.hk)

Content

Supplementary Notes 1-7

1. JSS (Jupiter-De-Spun-Sun) coordinate systems and the comparison with magnetic coordinate used for the footprint sketch.
2. Criteria for the Identification of Earth's, Saturn's and Jupiter's Cusps.
3. All examples of Jupiter's cusp.
4. Examples of Earth's and Saturn's cusp.
5. The Understanding of the Unexpected Duskside Location of Cusp.
6. The Velocity-Filter Effect of Ions Observed in Cusp.
7. Comparison to Similar Boundary Layers.

Supplementary Figures 1-16

Supplementary Tables 1-2

1. JSS (Jupiter-De-Spun-Sun) coordinate systems and the comparison with magnetic coordinate used for the footprint sketch.

In the Jupiter JSS coordinate system, x, y, z are defined as follows

$$\mathbf{Z} = \mathbf{J}_{\Omega} \tag{1}$$

Which is the unit vector of Jupiter spin axis. And

$$\mathbf{Y} = \mathbf{Z} \times \mathbf{R_{JS}} \tag{2}$$

$$\mathbf{X} = \mathbf{Y} \times \mathbf{Z} \tag{3}$$

in which $\mathbf{R_{JS}}$ is the unit vector of Jupiter to Sun (See Supplementary Fig. 1a). This system has the Z-axis aligned with Jupiter's spin axis but does not spin with the planet. JSS coordinate system are widely used in Jupiter space physics studies. The three components of the spherical coordinate system defined in the JSS coordinate system are as follows: The radial distance r is the Euclidean distance from the origin O to P; The azimuthal angle φ is the signed angle measured from the azimuth reference direction to the orthogonal projection of the line segment OP on the reference plane and counterclockwise is positive; The polar angle θ is the angle between the z direction and the line segment OP. See Supplementary Fig. 1b.

The schematics of a map of the magnetosphere and the location of Juno in the polar ionosphere are displayed in magnetic coordinates, whose center is the magnetic dipole axis (z-axis, as depicted in Supplementary Fig. 2a). In comparison, JSS coordinate used in the observation case is defined using Jupiter's spin axis. Since there is a tilt angle of ~9.5° between the spin axis and magnetic dipole axis in Jupiter, the spacecraft's magnetic latitude (MLat) and magnetic local time will continue to change periodically (see the bottom panel of Supplementary Fig. 2, using the data on April 15th, 2022 for example) during rotations. Therefore, the discrepancy explains why, within

the magnetic coordinate framework, Juno's footprint appears circular, even as its latitude and local time in JSS coordinates did not change significantly during the event.

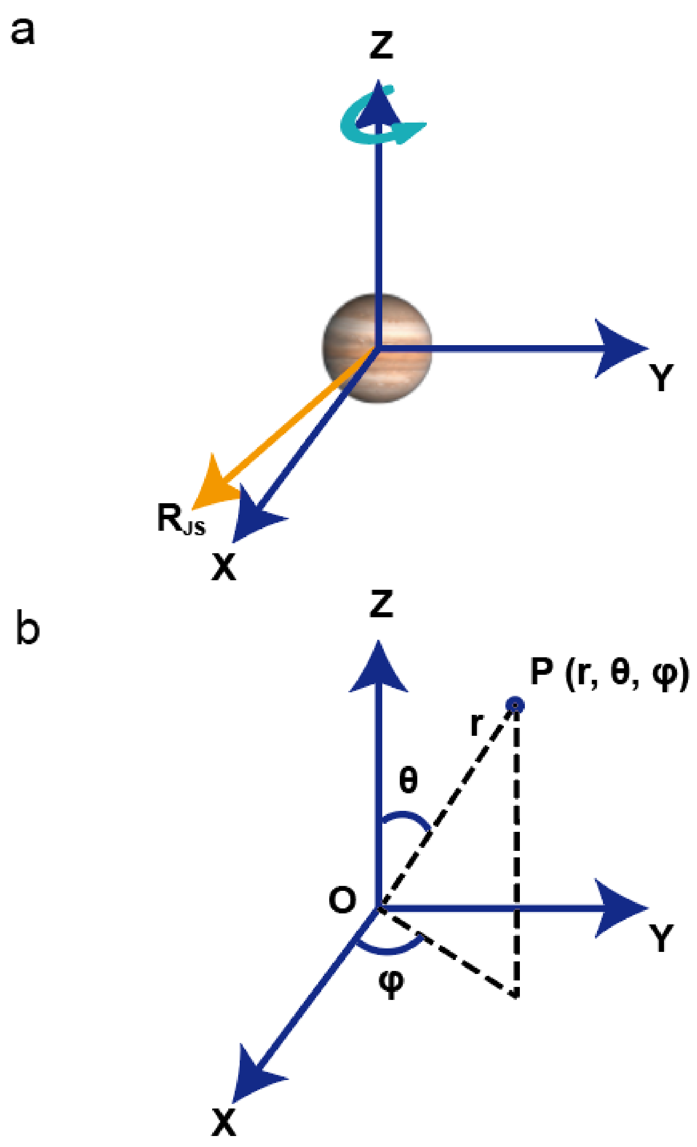

Supplementary Fig. 1. **Definition of coordinate systems used in the text.** (a) The definition of JSS (Jupiter-De-Spun-Sun) coordinates; (b) Definition of the spherical coordinate system (r, θ, φ) in JSS coordinates.

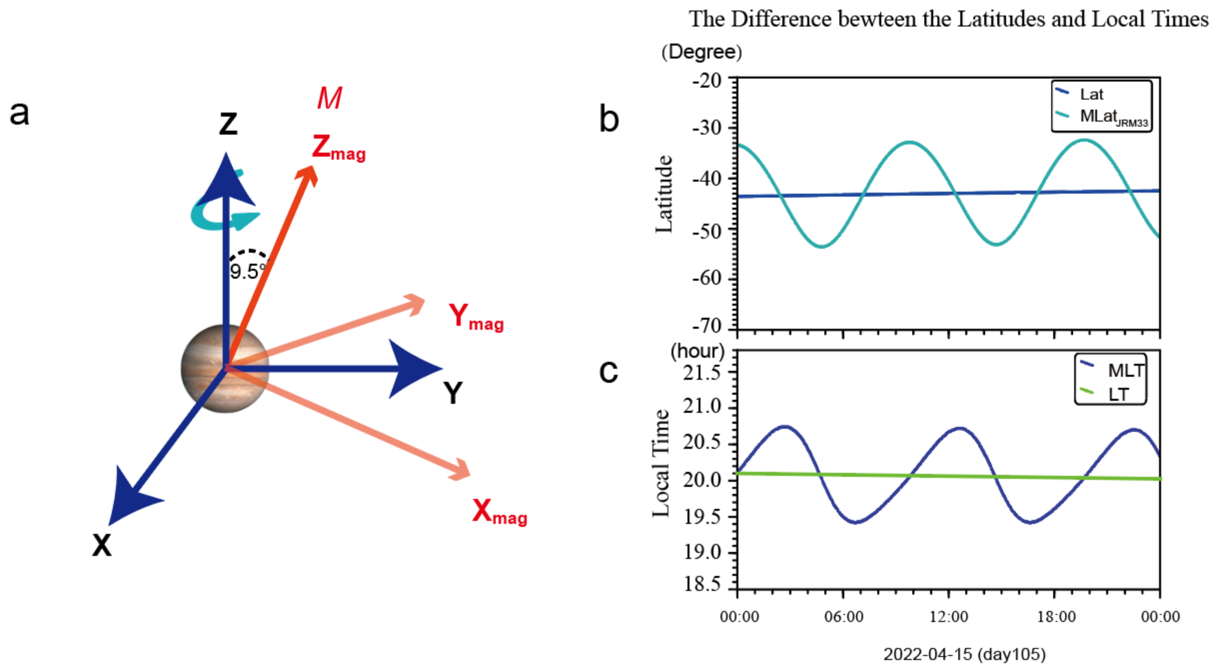

Supplementary Fig. 2. **The difference between the magnetic coordinate and the JSS coordinate.** (a) The definition of Jupiter magnetic coordinates; (b) Latitude (Lat) and Magnetic Latitude (Mlat) distribution on April 15th, 2022; (c) Local Time (LT) and Magnetic Local Time (MLT) distribution on April 15th, 2022.

2. Criteria for the Identification of Earth's, Saturn's and Jupiter's Cusps.

As shown in Supplementary Table 1, based on previous studies, the identification criteria for the cusps of Earth, Saturn, and Jupiter (inferred) are categorized into three levels of importance: level 1, level 2, and level 3, in descending order. Each criterion is elucidated and discussed sequentially below.

- Level 1 identification criteria for cusp (most important).

(a) Magnetosheath plasma features at high latitude inside the magnetosphere.

The magnetospheric cusp of a planet can be defined as part of the magnetosphere in the vicinity of the polar region at high magnetic latitudes/ invariant latitude, where a significant quantity of magnetosheath plasma is detected inside the magnetopause position[1–3]. Defined by its nature and position, a cusp is an region where magnetosheath plasma and momentum can enter the magnetosphere. Therefore, the spacecraft's high-latitude positioning and the detection of magnetosheath plasma constitute the primary criteria for cusp identification., high-latitude location and magnetosheath plasma/electron features are listed as ‘level 1’ identification criteria.

At Earth, magnetosheath particles entering the cusp typically include low energy ions within the 500 eV–5 keV range and soft electrons around 50 eV[4,5]. Low-energy magnetosheath-like electron distributions (10s ~ 100s eV enhancement) have been used to identify cusp events (in conjunction with the spacecraft's inside-magnetopause and high-latitude position) since in-situ observations in 1971[6,7], and this identification criterion has been used in cusp case reasearch for decades[1,4,8]. The distinction between Earth's cusp and other boundary layers has utilized the criterion of average electron spectrum enhancement, with levels $E_e < 220$ eV indicating magnetosheath-like conditions typical for the cusp, and enhancements from $220 < E_e < 600$ eV identifying other boundaries (e.g., cleft/LLBL[9,10]). Thus,magnetosheath-like electrons at high latitudes within the magnetosphere constitute a dependable criterion for identifying cusp regions, a methodology that has also been applied to the identification of Saturn's cusp[11–13] (~100 eV electron enhancement). In contrast, sheath-like ion features are more variable and less reliable for identification purposes than electrons, due to the velocity filtering effects linked with reconnection and the intermittent nature of cusp-related reconnection[14]. While electrons are also accelerated during reconnection, the velocity change is negligible[3].

(b) Magnetic depression.

It should be noted that magnetic depression features are also very important for identifying cusp at Earth[15–17]. The magnetic depression observed in the cusp region results from elevated plasma pressure and density due to the inflow of magnetosheath hot plasma, which is a magnetic turbulence feature that accompanies the magnetosheath inflow. In the cusp region, plasma thermal and magnetic pressures maintain a state of relative equilibrium. An increase in plasma thermal pressure leads to a significant reduction in magnetic pressure within the outer cusp, ensuring the maintenance of pressure equilibrium with the adjacent magnetospheric regions[18,19]. Lavraud et al.[14] statistically demonstrated the presence of a diamagnetic cavity within the cusp region.

However, magnetic depression features are not always present in Saturn cusp events. Magnetic depression features are inconsistently observed in Saturn's cusp events. While some cases exhibit marked magnetic depressions akin to Earth's, others display weak or no magnetic field disturbances (e.g., see Figure 4, overview of Jan 2007 in Arridge et al.[13]). Jasinski et al.[20] reported that only two out of five winter cusp events exhibited magnetic depressions. Similarly, analysis of Juno data indicates that Jupiter's cusp events, as displayed in text S3, largely lack magnetic depression features. The absence of this feature for some cusp cases of giant planets remains unresolved. A plausible explanation considers the correlation between the plasma thermal pressure in the cusp and the solar wind dynamic pressure surrounding the planet. The solar wind dynamic pressure at the orbits of the giant planets (Jupiter at 5 AU, Saturn at 9 AU) is significantly lower than at Earth, while their magnetic field strengths in the cusp regions are comparable (10-100 nT). Consequently, the plasma from the magnetosheaths of these giant planets enters the cusp with generally lower thermal pressure relative to magnetic pressure, not necessarily inducing magnetic depression. This relationship is attributed to the solar wind's thermal pressure being minor compared to its ram pressure, with the latter being a major contributor to magnetosheath pressure[14],

which, in turn, influences the thermal pressure of plasma entering the cusp. According to Spreiter et al.[21], magnetosheath pressure, a linear function of the solar wind Mach number squared (M^2), can be approximated as proportional to V_{sw}^2, and magnetosheath density correlates with solar wind density. Therefore, within reasonable Mach number ranges, magnetosheath pressure correlates with ρV_{SW}^2. Lavraud et al.[14] conducted a statistical analysis on the ratio of plasma thermal pressure to solar wind dynamic pressure inside and outside the cusp, finding the magnitudes to be very comparable (see Figure 6b in it). Furthermore, Zhou et al.[18] and Jasinski et al.[20] statistically examined the correlation between cusp magnetic depression and solar wind dynamic pressure for Earth and Saturn, respectively, with the latter utilizing the Michigan Solar Wind Model. Their findings indicated that higher solar wind dynamic pressures lead to more pronounced cusp magnetic depressions. And studies on Mercury[22–25] have shown magnetic depression to be stronger than at Earth. These investigations collectively highlight the significant influence of the solar wind environment on cusp magnetic depression phenomena.

In summary, based on the cusp's definition and extensive observational reports, the presence of magnetosheath-like electron features at high latitudes well within the magnetopause is determined as the paramount criterion for identifying Jupiter's cusp. Magnetic depression, considered a key identification criterion for Earth, is not deemed a necessary feature for the giant planets. Therefore, it is classified as a Level 2 criterion.

- Level 2 identification criteria for cusp (less important but helpful).

The cusp, being the region where magnetosheath plasma and momentum enter the magnetosphere, exhibits plasma characteristics that are significantly influenced by magnetic reconnection. At Earth, the cusp's ion characteristics are affected by the magnetic reconnection process occurring at low latitude[26,27] or in high-laitutude lobe region[28,29]. Numerous studies have shown that, within Earth's

cusp, ions often display dispersion[26,27,30] or "reversed" dispersion patterns[28,31], which have been used as an important feature in identify cusp. These patterns arise from the velocity filtering effect linked to acceleration during magnetic reconnection. The E×B drift causes high-energy ions to reach a given altitude before their slower counterparts, which arrive at the same altitude but at a different location, such as latitude. This time-of-flight effect, in combination with transverse convection, results in the well-known ion dispersion[26,32]. Different dispersion characteristics correspond to different solar wind conditions and reconnection locations[30,31]. Normal and reversed ion dispersion features have also been used to identify Saturn's cusp[11–13].

However, at Earth, due to the cusp's dynamic nature and the sporadic occurrence of magnetic reconnection, plasma flows within the cusp often exhibit intermittent features[5,33–35], which are affected by the solar wind and the spacecraft's position. Consequently, not all observations within the cusp show clear ion dispersion. Reports on Saturn's cusp indicate similar findings[12,13]. Thus, ion dispersion is classified as a Level 2 criterion, underlining its significance yet acknowledging it is not necessity for cusp identification. Furthermore, as outlined in section (b) regarding Level 1 criteria, magnetic depressions are also categorized under Level 2.

- Level 3 identification criteria for cusp (not necessary but can help).

At Earth, enhanced plasma waves ranging from 1 to 100s Hz—where the upper limit is the electron cyclotron frequency—are often utilized in conjunction with magnetic fields and plasma flows to identify the cusp[36–38]. These auroral-like hiss waves are believed to consist of whistler mode emissions[38,39] and are thought to be produced by a Cerenkov radiation mechanism[40]. Similarly, auroral hiss has been detected in the very high-latitude magnetosphere of Jupiter[41], akin to the hiss observed over Earth's auroral zones . It has been proposed that these emissions were generated by field-aligned electron beams[41], which may arise from reconnection-related processes. Stone et al.[42]

considered observations of enhanced auroral hiss near 100 Hz as potential indicators of Jupiter's cusp. Although less extensively studied at Saturn, it has also shown associations between auroral hiss and cusp regions. Auroral hiss Enhancements around 100 Hz have been observed within parts of the cusp, and in adjacent areas[11,13].

In summary, auroral hiss can be used to assist in the identification of cusp, which can be generated by field-aligned electron beams produced by cusp-associated reconnection processes. However due to the sporadic nature of reconnection and the complexity of the cusp environment, auroral hiss is not consistently present in cusp regions, and thus it is classified as a Level 3 criterion, which can help identify cusp but is not necessary.

Similarly, field-aligned electron pitch angle features (enhancements near 0 and 180°), linked to reconnection processes, can facilitate the identification of the cusp on both Earth and Saturn[3,11,13]. However, there may be other properties of the electron pitch angle in the cusp, for example, enhancements around 90° due to local acceleration related to gradients in reconnected quasi-potential[43] and wave-particle interactions[44,45]. Consequently, given the variable nature of electron pitch angle data within the cusp, this feature is also classified as a Level 3 criterion—helpful for identification but not very important.

Identification of Cusp	Level 1 (key)				Level 2		Level 3		
Earth	Inside Magnet-opause	High Latitude Location	Magnetoshe-ath-like Electron	Mag-netic Depr-ession	Reconnetion -Related Ion Dipersion	-	Whistler -mode Hiss Wave	Electron Pitch Angle Features	Other
Saturn	Inside Magnet-opause	High Latitude Location	Magnetoshe-ath-like Electron	-	Reconnetion -Related Ion Dipersion	Magn-etic Depres sion	Whistler -mode Hiss Wave	Electron Pitch Angle Features	Other
Jupiter (inferred)	Inside Magnet-opause	High Latitude Location	Magnetoshe-ath-like Electron	-	Reconnetion -Related Ion Dipersion	Magn-etic Depres sion	Whistler -mode Hiss Wave	Electron Pitch Angle Features	Other

Supplementary Table 1. Summary comparison of cusp identification criteria for Earth, Saturn and inferences of identification criteria for Jupiter's cusp.

3. All examples of Jupiter's cusp.

This section summaries the features of all cusp cases in this study (Supplementary Fig. 3-8), all well meeting the Level 1 criteria for Jupiter. Moreover, the majority of these events also exhibit characteristics outlined in Levels 2 as listed in Supplementary Table 1, 2 (e.g., ion dispersion and hiss wave). Furthermore, all events also exhibit enhanced auroral hiss characteristics outlined in Level 3 as listed in Supplementary Table 1, 2. The compliance of all events with the identification criteria is detailed in Supplementary Table 2.

Identification of Cusp	Level 1 (key)			Level 2		Level 3		
Jupiter (inferred)	Inside Magneto-pause	High Latitude Location	Magnetosheath-like Plasma/Electron	Reconnetion-Related Ion Dipersion	Magnetic Depression	Whistler-mode Hiss Wave	Electron Pitch Angle Features	Other
Jovian cusp case 1	√	√	√	√	-	√	-	-
Jovian cusp case 2	√	√	√	√	-	√	√	-
Jovian cusp case 3	√	√	√	√	-	√	-	-
Jovian cusp case 4	√	√	√	√	-	√	-	-
Jovian cusp case 5	√	√	√	√	-	√	-	-
Jovian cusp case 6	√	√	√	√	-	√	√	-

Supplementary Table 2. The list which outlines how the criteria are met for the extended cusp cases. Due to the poor coverage of electron pitch angle data across most event observations, features such as field-aligned electrons are not discernible in the majority of events.

All cusp cases in this study all show clear magnetosheath-like electron features (panel c in Supplementary Fig. 3-8) labeled by red dashed lines in Supplementary Fig. 3-8. And all cases were observed in high invariant latitude and the quite magnetic field indicates that the spacecraft was well inside the magnetopause (shown in panel h and label information in Supplementary Fig. 3-8). So in summary, all 6 cases well meet the Level 1 criteria, which is the mandatory identification criteria for cusp events. Moreover, case 1 to case 5 show clear ion dispersion within cusp regions. For case 6 due to the short duration of the cusp crossing, only 40 minutes (16:42 to 17:25 UT on April 15, 2022), the ion dispersion is not as pronounced as the other events, which is why it is replaced by other, more typical events in the main text.

Cases 1, 3, 5, and 6 (panel d in Supplementary Fig. 3-8) exhibit normal ion dispersion, whereas Case 2 presents reversed ion dispersion (panel d in Supplementary Fig. 3-8). Case 4 displays multiple ion dispersion features (panel d in Supplementary Fig. 3-8) simultaneously, likely due to complex solar wind conditions and the dynamics of magnetopause reconnection. So Level 2 criteria are well met for all cases. In cases 2, 3, and 4, the proton and heavy ion energy spectra within the cusp regions reveal predominantly single species of protons and heavy ions, with the latter being almost absent, as shown in panels d and e of Supplementary Fig. 4-6. In comparison, the cusp regions of cases 1, 5, and 6 exhibit two distinct plasma components. One component is similar to those observed in cases 2, 3, and 4, while the other shows a significant enhancement of the proton energy spectrum at a few 100s eV and a dual peak in the heavy ion energy spectrum at

~10000 eV (and ~1000 eV), as illustrated in panels d and e of Supplementary Fig. 3, 7, 8. This variation highlights the complex nature of the cusp, which may reflect interactions with different flux tubes as the spacecraft enters the cusp.

All cases do not exhibit significant magnetic depressions within the cusp region. Another possible explanation for the lack of magnetic depressions is the local time all cases occur close to the dusk side, away from local noon. Statistical studies of the Earth's cusp have demonstrated that the closer the local time is to noon, the more pronounced the magnetic depression[18]. Similarly, cusp events at Saturn showing magnetic depression features were observed near noon[13,20].

Enhanced auroral hiss waves near 100 Hz were detected in the cusp region during the flight for all cases, as depicted in panel g of Supplementary Fig. 3-8. Specifically, the enhancement of auroral hiss in cases 3 and 5 is tightly confined to the cusp region. In contrast, auroral hiss in the other cases were also observed in the cusp-adjacent magnetosphere or boundary/mixed layers. This broader distribution may be attributable to the presence of field-aligned electron beams in these adjacent regions, or it could be related to the generation and propagation of auroral hiss, which depend on resonance cones that are angled relative to the background magnetic field. This angle allows the source region adjacent to the cusp to be detectable.

The identification of field-aligned electron features is challenging due to the absence of significant electron data near the pitch angles of 0 and 180 degrees in most events. For instance, the electron pitch angle distributions in cases 1, 3, 4, and 5 have poor coverage, as shown in panel f of Supplementary Fig. 3, 5-7, making it challenging to determine from the normalized electron pitch angle data whether the enhancements in pitch angle are aligned with the magnetic field. In cases 2 and 6, although the electron pitch angle data also exhibit poor coverage, it is possible to identify pitch angle flux enhancements greater than 150° and less than 30°.

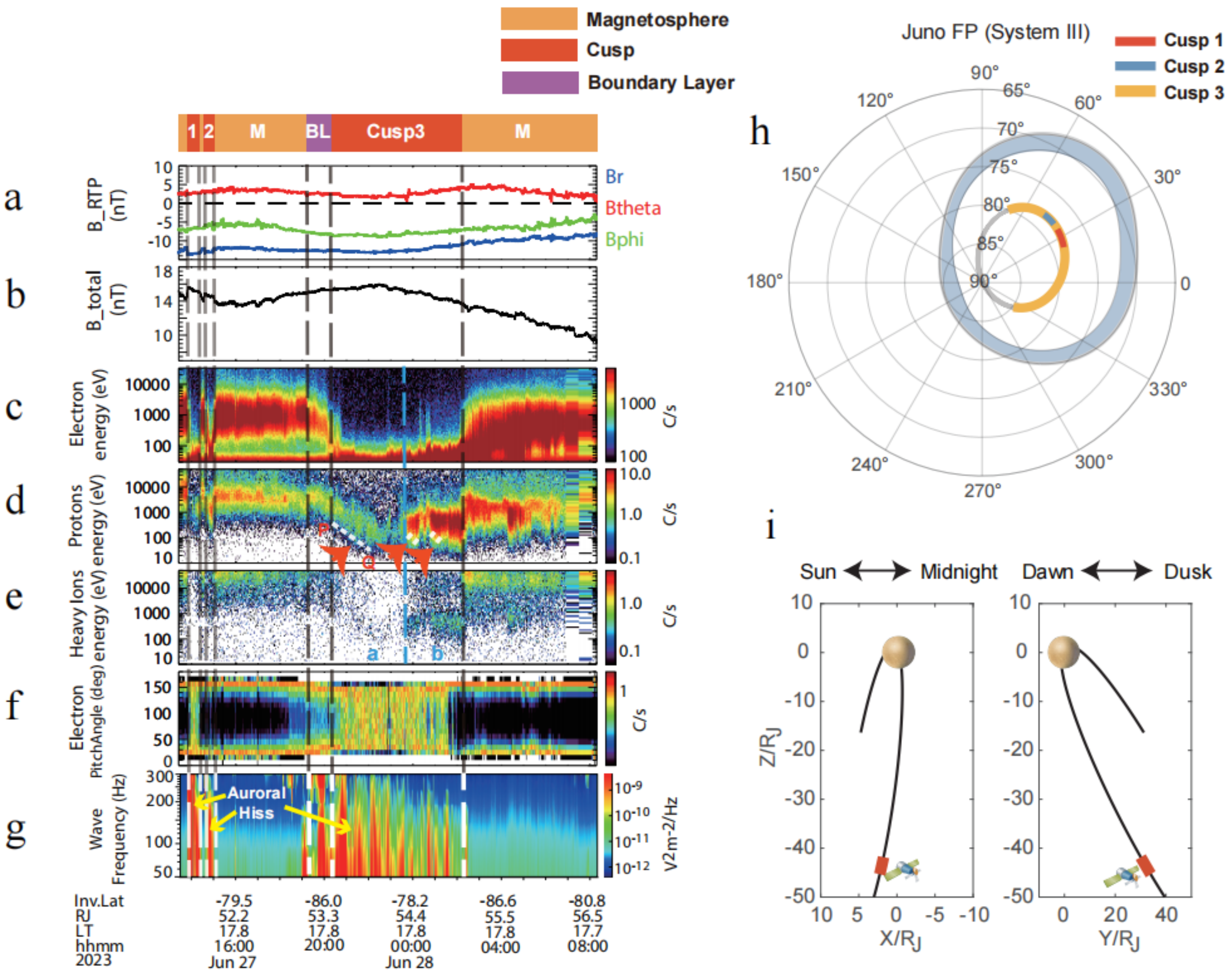

Supplementary Fig. 3. **Cusp example 1 showing clear typical ion dispersion (panel d).** (a) R-Theta-Phi magnetic field components in JSS (Jupiter-De-Spun-Sun) coordinate; (b) The total magnetic field strength; (c) The electron energy spectrogram; Ion Energy spectrogram for protons (d) and heavy ions (e), where heavy ions represent ions with m/q in the range of 5 and 64; (f) Pitch angle distribution for electrons which is normalized at each time unit within energy ranges of 0.3 to 32 keV; (g) Plasma wave observations in the frequency range 50 to 300 Hz. The different regions that the spacecraft passes through are marked with different colors at the top and separated by dashed lines. 'M' is the magnetosphere, 'C' is the cusp, 'BL' is the boundary layern. The red arrows and white dashed line in panel (d) show the dispersion. The yellow arrows in panel (g) indicate the

enhanced auroral hiss features. The blue dashed line demarcates the cusp into two regions, labeled as 'a' and 'b', each characterized by different plasma properties. (h) Traced distribution of spacecraft footprints before and after cusp observation in Left-Handed system III coordinates. The red regions are the main ovals, and the colored lines are the Juno footprint trajectories before and after the cusp region observation (June 26th to April 28th, 2023). (i) The position of the spacecraft around cusp observations, red lines representing the time range of the cusp case.

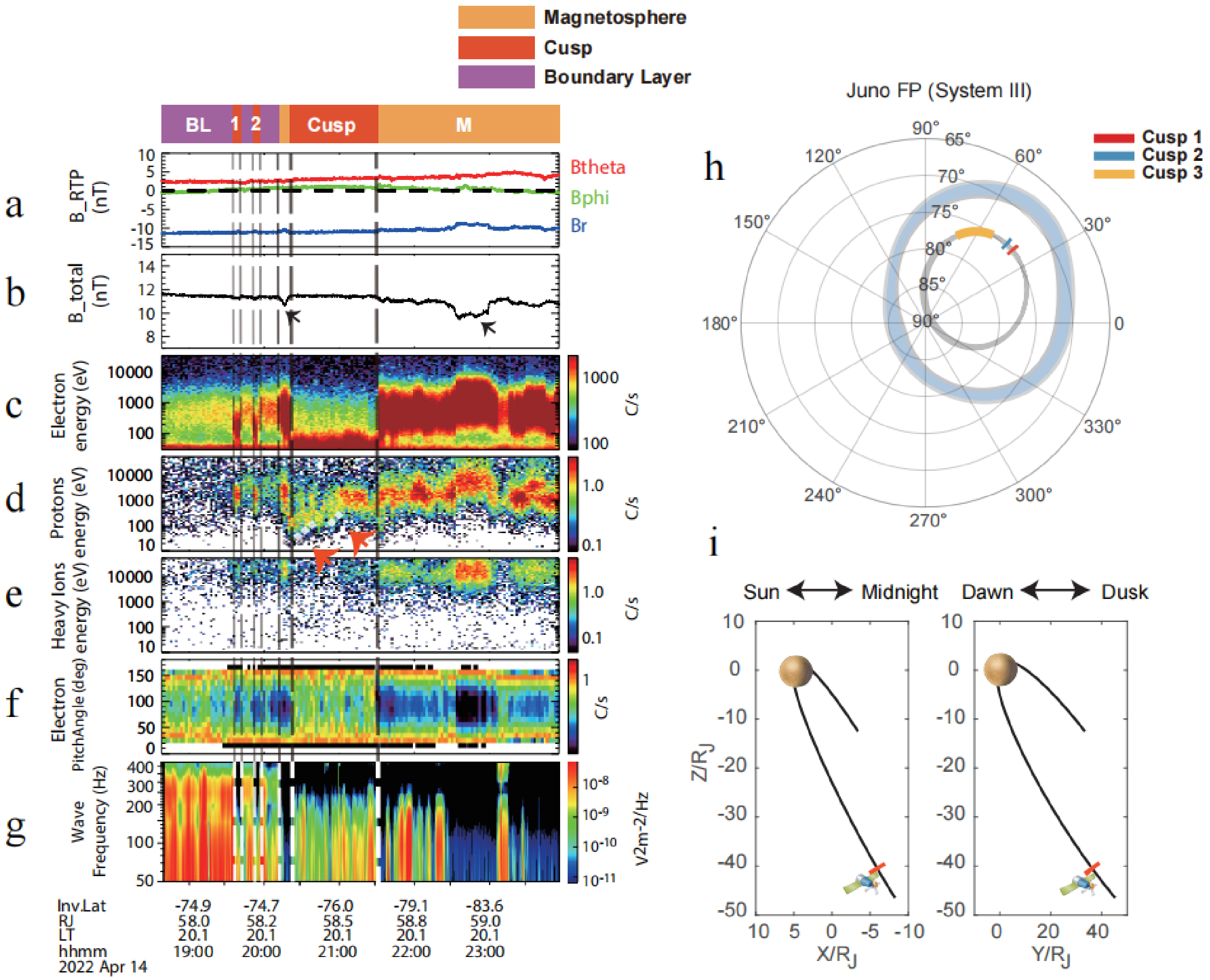

Supplementary Fig. 4. Cusp example 2 showing clear reversed ion dispersion (panel d). Panel a-i are arranged in the same format as Supplementary Fig. 3.

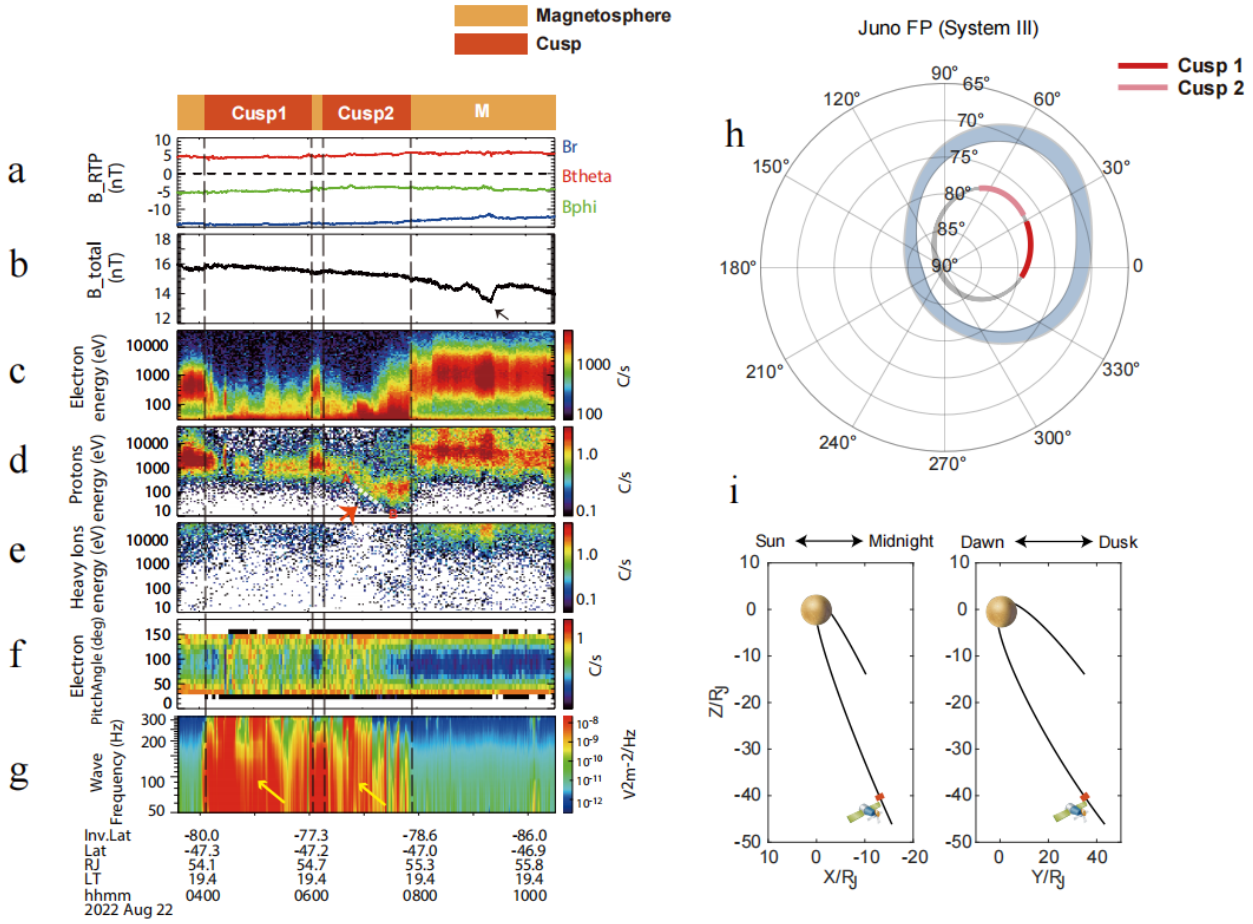

Supplementary Fig. 5. **Cusp example 3 showing clear ion dispersion (panel d).** Panel a-i are arranged in the same format as Supplementary Fig. 3.

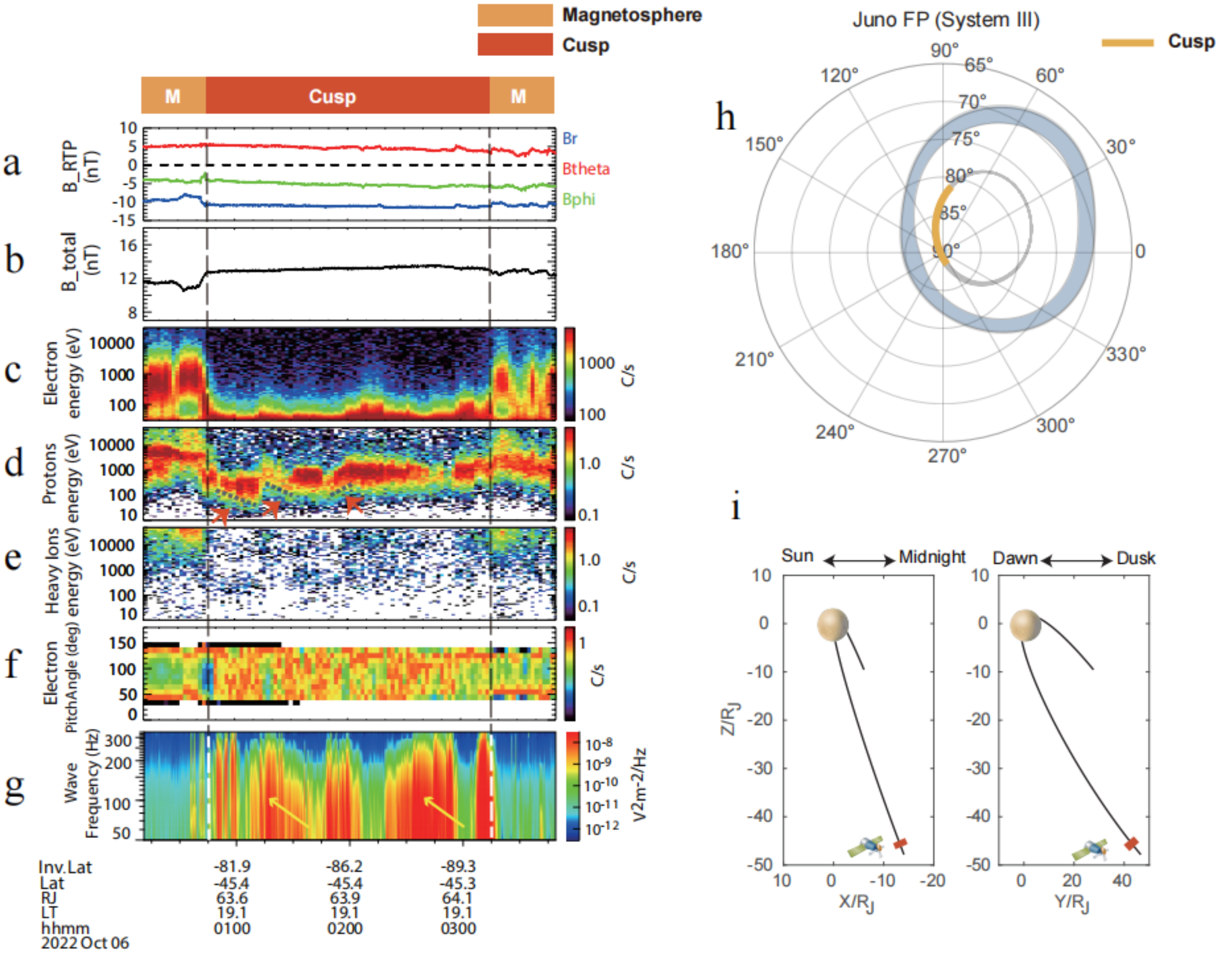

Supplementary Fig. 6. Cusp example 4 showing clear ion dispersion (panel d). Panel a-i are arranged in the same format as Supplementary Fig. 3.

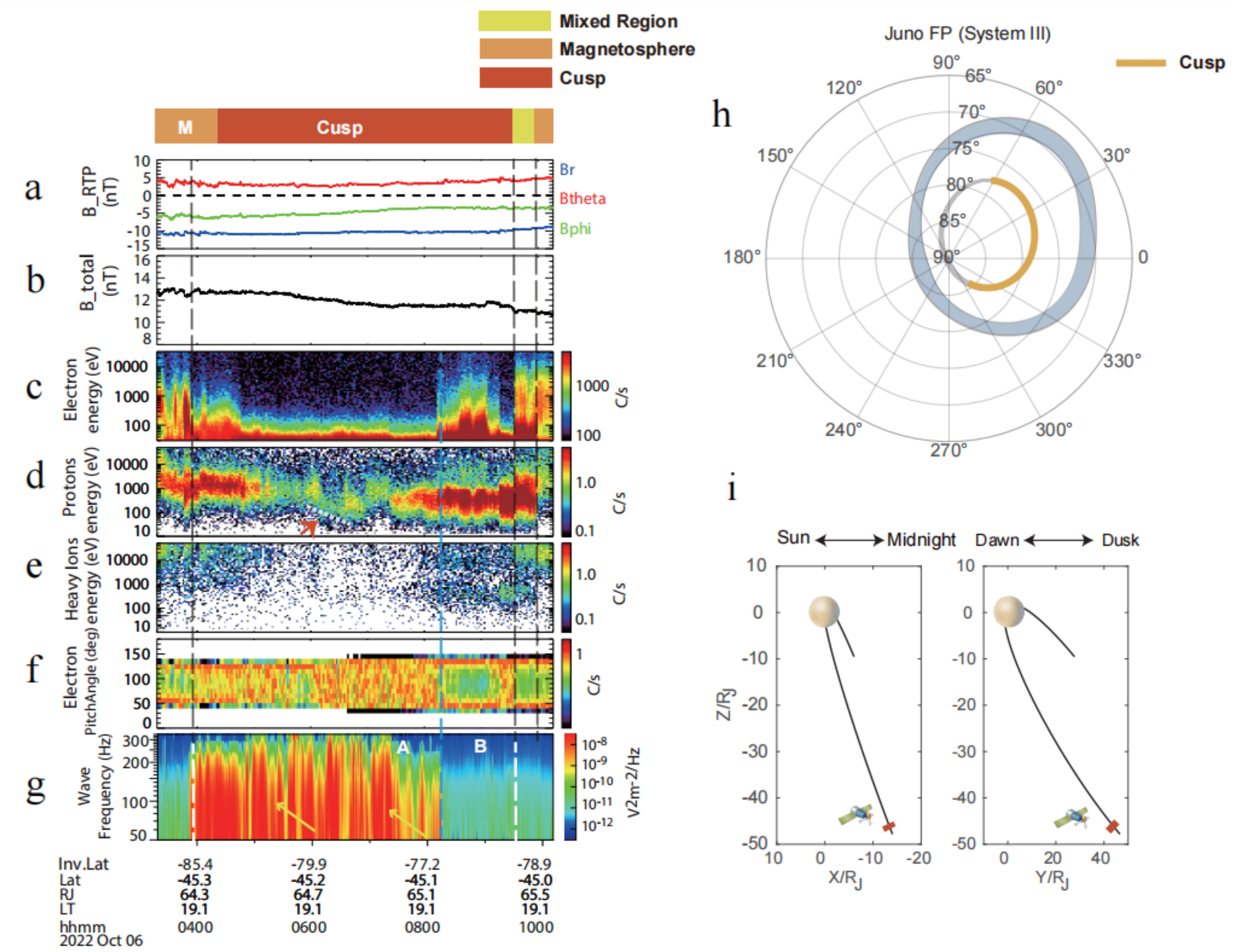

Supplementary Fig. 7. Cusp example 5 showing clear ion dispersion (panel d). Panel a-i are arranged in the same format as Supplementary Fig. 3. 'Mixed region' is identified by the apparent mixture of the proton and heavy ion components from both the magnetosphere and the cusp regions. The blue dashed lines separating regions A and B within the cusp distinguish regions with strong auroral hiss enhancement from those without. The absence of auroral hiss in area B may be linked to the lack of detection of reconnection-correlated field-aligned electron beams. E.g., panel d reveals that the protons in region B do not exhibit significant dispersion.

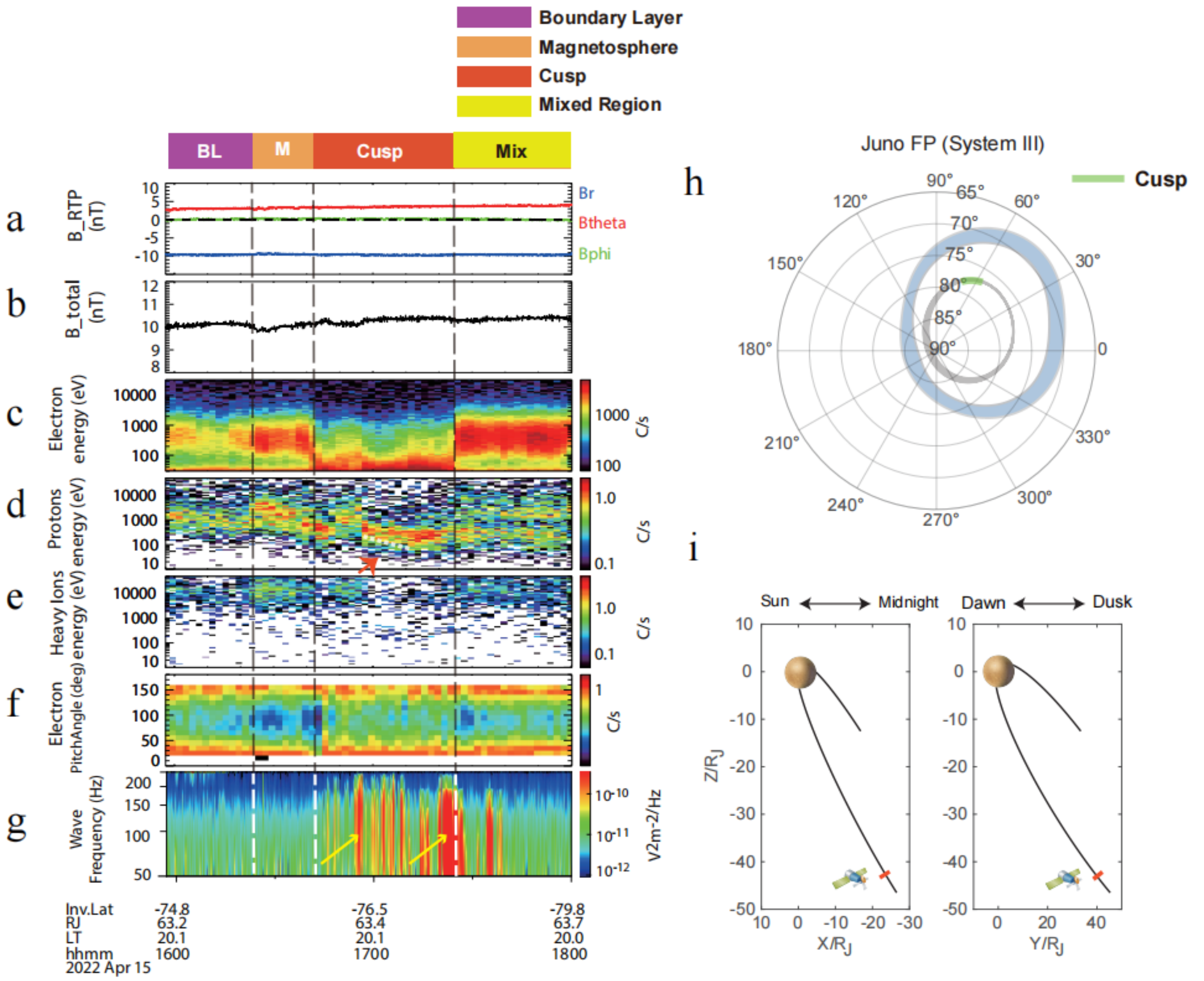

Supplementary Fig. 8. Cusp example 6 showing ion dispersion (panel d). Panel a-i are arranged in the same format as Supplementary Fig. 3. ‘Mixed region’ is identified by the apparent mixture of the protons components from both the magnetosphere and the cusp regions.

4. Examples of Earth's and Saturn's cusp.

Supplementary Fig. 9 shows the examples of the Earth’s and Saturn’s cusp. The data sets presented for Earth’s cusp were obtained by the FGM[46] , PEACE[47] , CIS[48], WHISPER[49] and instruments on board Cluster. The data sets presented for Saturn’s cusp were obtained by the MAG[50], CAPS[51] instruments on board Cassini.

Both in Earth's and Saturn's cusp regions, the electron energy spectra are magnetosheath-like. And Supplementary Fig. 9d and Supplementary Fig. 9i show the ion dispersion features in the cusp, which are very similar to the Jupiter observations in the main text. The similarities of the electron and ion features imply that similar cusp microphysics exist at different planets.

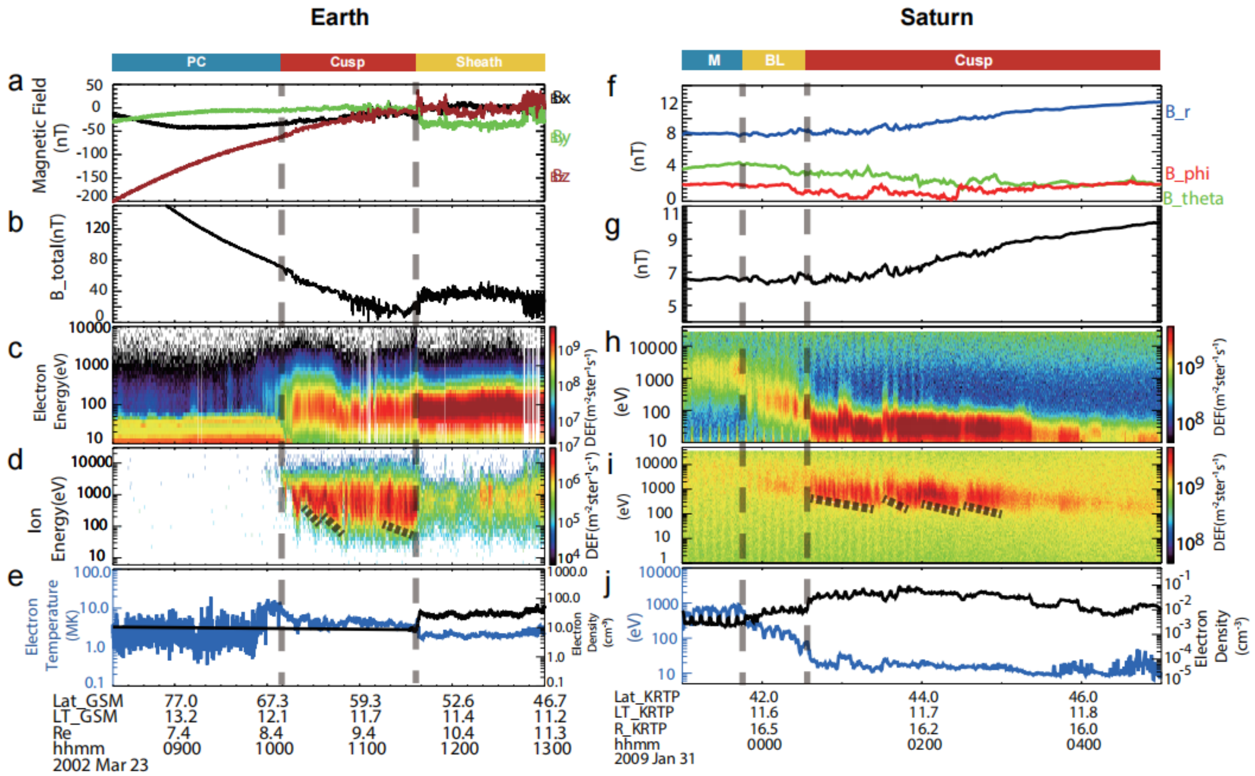

Supplementary Fig. 9. **Cusp case at Earth observed by Cluster and Saturn observed by Cassini.** The magnetic field (a) x-y-z components at Earth in GSM coordinate system and (f) R-Theta-Phi components at Saturn in KRTP coordinates; The magnitude of magnetic field at (b) Earth and (g) Saturn; The electron spectrum for (c) Earth and (h) Sartun; The ion spectrum for (d) Earth and (i) Sartun; The electron temperature and electron density for (e) Earth and (j) Sartun. The black dashed line marks the ion dispersion. Different regions are labeled at the top. 'PC' represents the polar cap, 'M' represents the magnetosphere and BL 'represents the boundary layer.

5. The Understanding of the Unexpected Duskside Location of Cusp.

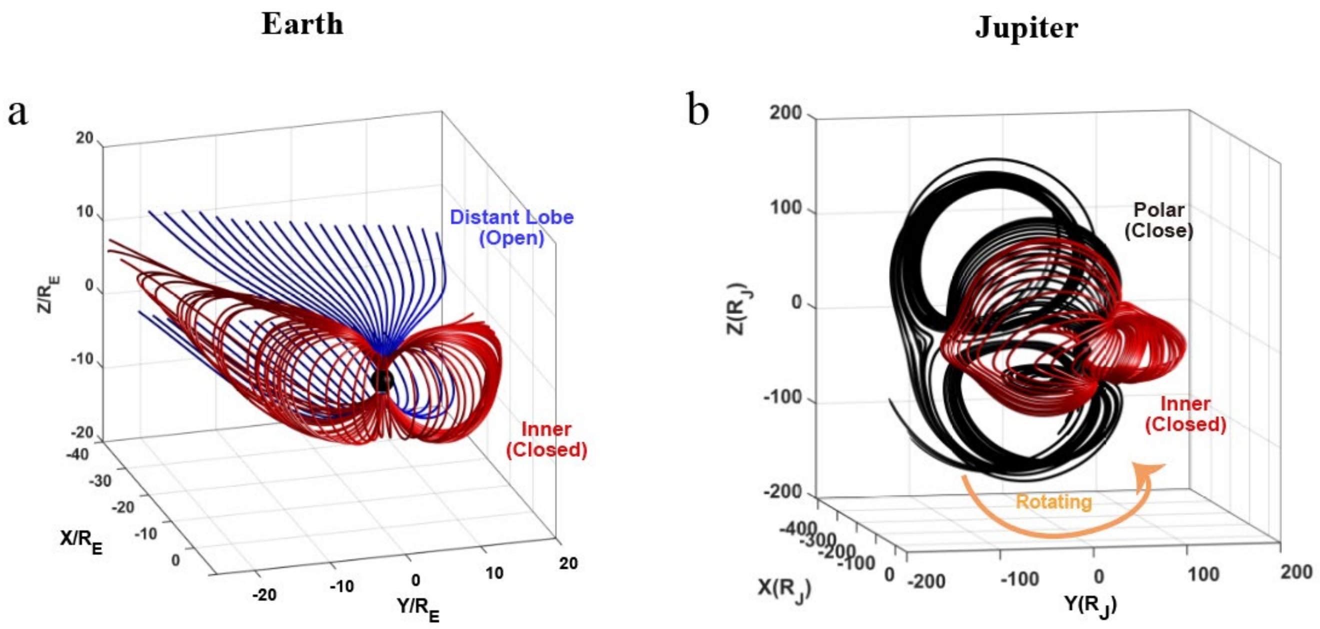

Supplementary Fig. 10. Comparison of Earth's and Jupiter's magnetic configuration using Chen et al. simulation results[52]. (a) Earth's and (b) Jupiter's magnetic configuration. The former exhibits dawn-dusk symmetry, whereas the latter demonstrates dawn-dusk asymmetry attributed to the effects of rapid rotation.

The auroral morphologies observed on Jupiter, differing from those on Earth and Saturn, suggest an unusual magnetic field configuration and cusp distribution that sets Jupiter apart from the traditional auroral pictures associated with Earth. Terrestrial auroral emissions are generated by the precipitation of energetic particles along magnetic field lines[53,54]. These emissions form an auroral oval that encircles the magnetic pole. In contrast, although Saturn's magnetic fields are also predominantly dipolar, its auroral emissions display significant variations with complex morphologies[55–57], exhibiting features characteristic of both Earth-like and Jupiter-like structures. At Jupiter, the aurora present a distinctly different morphology and evolutionary pattern[58–60],

characterized by a highly dynamic yet generally bright polar cap aurora and contrasting dark "polar collar" regions.

Zhang et al.[61] provide the only study to date that comprehensively explains these different auroral morphologies on Jupiter, whose insights into Jupiter's magnetic field structure have also established a crucial foundation for understanding the unusual cusp locations observed in this study. The formation and different configuration of Jupiter's cusp can potentially be attributed to the solar wind environment around Jupiter and the asymmetrical configuration of Jupiter's rapidly rotating magnetosphere. Additionally, the strong centrifugal force exerted during Jupiter's rapid rotation causes its magnetospheric configuration to appear "flatter," resulting in a greater presence of topological eastward/westward components, as shown in Supplementary Fig. 10. This flattened structure is suggested to be more sensitive to east-west solar wind-induced magnetic reconnection, as shown in Supplementary Fig. 11.

Furthermore, previous research indicates that both the interplanetary magnetic field (IMF) azimuthal angle[62] and the clock angle[63,64] around Jupiter are predominantly around ±90°, suggesting that the solar wind near Jupiter is primarily east-west oriented (B_y component dominant). This orientation facilitates a unique coupling of the solar wind to Jupiter's magnetosphere, which exhibits a distinct magnetic field configuration, as depicted in Supplementary Fig. 11. This configuration allows for the existence of open magnetic field lines coupled to the solar wind on the dusk side inside the magnetosphere. It is within this portion of the magnetosphere that the cusp region can be observed, as shown in the boxes in Supplementary Fig. 11a and 11b.

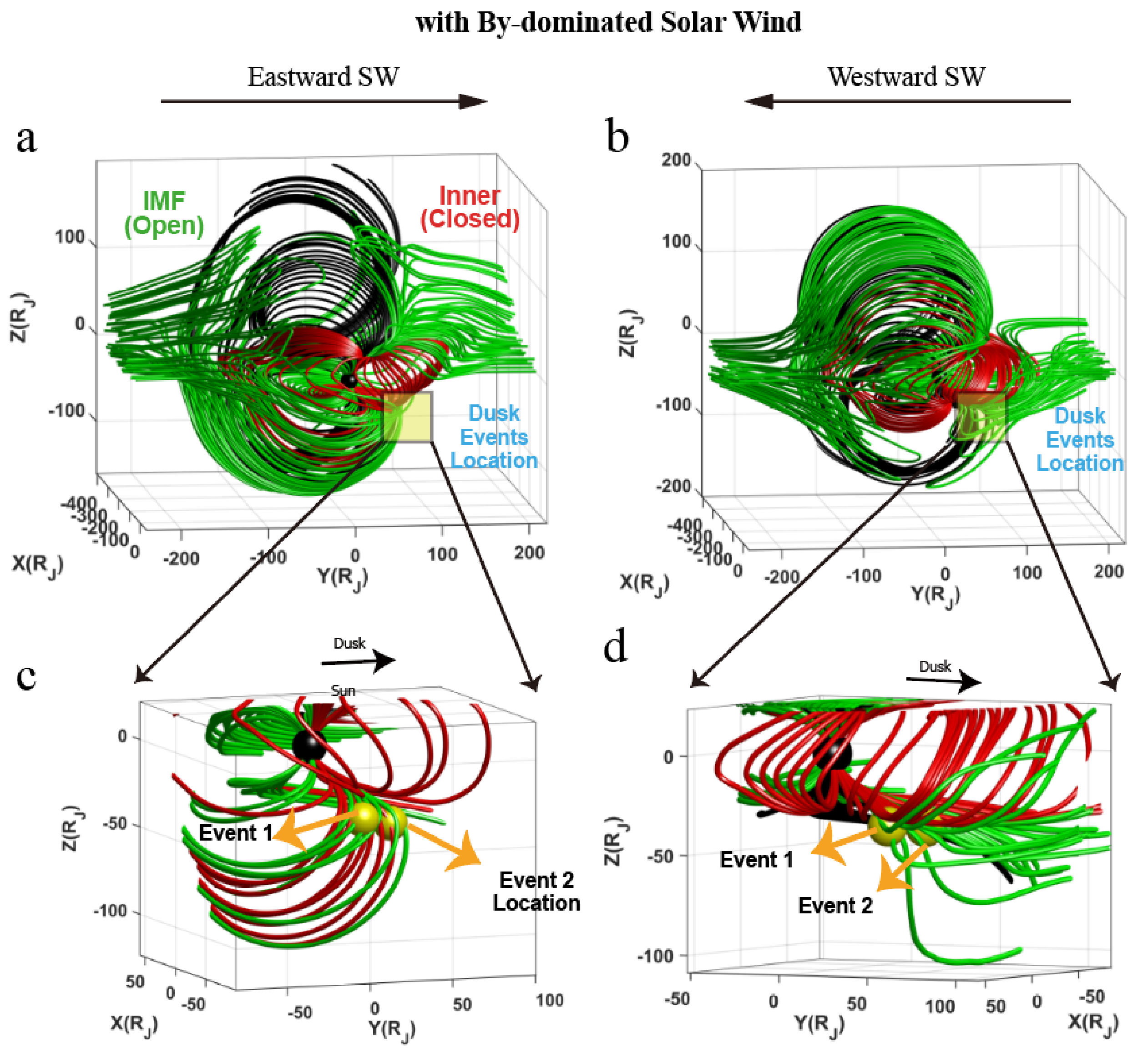

Supplementary Fig. 11. Jupiter's magnetic configuration under different typical olar wind conditions. (a) Under eastward / (b) westward solar wind conditions. (c) and (d) depict the zoomed-in magnetospheric structure at the location of the cusp events under different solar wind conditions using Chen et al. simulation results[52]**.** The yellow spheres, labeled "event 1" and "event 2," denote the positions of the spacecraft during the pre-dusk cusp case 1 (Supplementary Fig. 3) and the post-dusk cusp case 2 (Supplementary Fig. 4), which are discussed in detail in the main text.

6. The Velocity-Filter Effect of Ions Observed in Cusp.

This section provides brief discussions of the velocity-filter effect of ions observed in the cusp; for more detailed information see Smith and Lockwood[3] and the references therein. When the spacecraft and magnetic field line convection move in the same direction, three scenarios emerge: (1) The spacecraft's velocity $|V_{sc}|$ surpasses that of plasma convection $|V_{conv}|$, as illustrated in Supplementary Fig. 12a. Under these conditions, the spacecraft will sequentially pass through magnetic field lines A, B, and C, owing to its higher speed. Given that a recent magnetic reconnection has occurred near magnetic field line A, the spacecraft is likely to detect fast, high-energy ions first. Slower, low-energy ions produced by the reconnection will subsequently be detected on magnetic field lines B and C which underwent reconnection earlier. As time progresses, high-energy ions on lines B and C will dissipate due to bouncing motion. Consequently, in Supplementary Fig. 12a, the farther the magnetic field lines are from the reconnection point, the lower the ion energy detected by the spacecraft will be. As a result, the spacecraft is expected to observe normal ion dispersion[26,30] as shown in Supplementary Fig. 12e.

(2) $|V_{sc}| \sim |V_{conv}|$ as shown in Supplementary Fig. 12b. In this situation, given that the spacecraft's speed matches that of the magnetic field line convection, the spacecraft remains aligned with magnetic field line A throughout its trajectory. As a result, the spacecraft is anticipated to first detect fast, high-energy ions and subsequently encounter slower, lower-energy ions. Accordingly, the spacecraft is expected to observe a pattern of normal ion dispersion[26,30] as shown in Supplementary Fig. 12f.

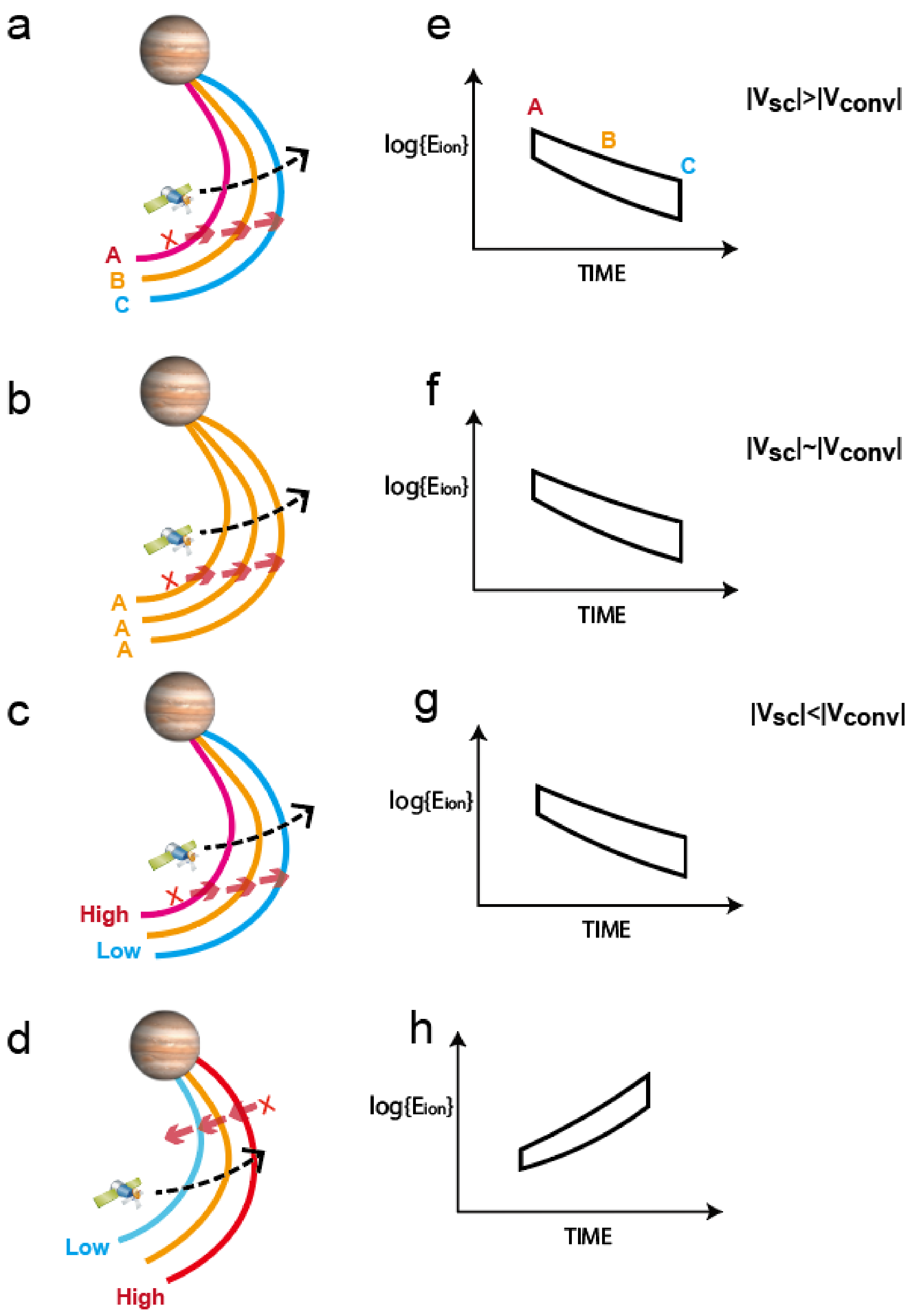

Supplementary Fig. 12. The schematic of velocity-filter effect of ions observed in cusp for different situations. (a, b, c) Schematic when Juno moves in the same direction of magnetic field line convection motion; (d) Schematic when Juno moves against the convection direction of magnetic field line; (e) The expected ion energy distribution detected by the spacecraft in the case

corresponding to (a) and when the spacecraft velocity is greater than the convection velocity; (f) The expected ion energy distribution corresponding to (b) and when the spacecraft velocity is approximately equal to the convection velocity; (g) The expected ion energy distribution corresponding to (c) and when the spacecraft velocity is less than the convection velocity. (h) The expected reversed ion dispersion distribution corresponding to (d). The 'X' flag marks the reconnection point. Red arrows and black dashed arrows indicate the convection direction of the magnetic field lines and the direction of the spacecraft's motion, respectively. The red, orange, and blue colors of the magnetic field lines indicate the magnetic lines from near to far from the reconnection point, respectively. The red indicates that a fresh magnetic reconnection is occurring and the blue indicates the opposite.

(3) $|V_{sc}| < |V_{conv}|$ shown in Supplementary Fig. 12c. In the third case, a newly reconnected magnetic line moving faster than the spacecraft will sweep over the spacecraft, populated with high-energy ions. Thus, the spacecraft will initially detect these high-energy ions. Subsequently, the spacecraft will be swept over by multiple additional magnetic field lines. As time elapses after reconnection and/or the spacecraft moves away from the reconnection site, lower-energy ions will be detected by the spacecraft. Therefore a pattern of normal ion dispersion[26,30] is generally expected (Supplementary Fig. 12g).

However, when the spacecraft and magnetic line convection move against each other, irrespective of their respective speeds, the spacecraft and magnetic field lines are constantly in relative motion. As illustrated in Supplementary Fig. 12d, in contrast to the scenario depicted in Supplementary Fig. 12c, the spacecraft will initially traverse older reconnected magnetic field lines carrying low-energy ions, followed by newly-reconnected field lines with high-energy ions. Consequently, the

spacecraft is anticipated to observe a reversed ion dispersion[30,31], as shown in Supplementary Fig. 12h.

7. Comparison to Similar Boundary Layers.

This section engages in a comparative analysis between boundary layers that exhibit similarities in particle properties with those in the cusp region and the cusp itself. These boundary layers encompass both the magnetopause boundary layer and the plasma sheet boundary layer[65] (PSBL). In order to reasonably compare all the events, all colorbars of Supplementary Fig. 13 to 16 are set to be identical.

(1) **The comparison between magnetopause boundary layer (Supplementary Fig. 13) and cusp (Supplementary Fig. 16).**

Both the cusp and the magnetopause boundary layer exhibit similarities in their enhanced low-energy electron energy spectrum (~100 eV), manifesting as magnetosheath-like electron distributions. However, the intensity of the low-energy electron flux in the magnetopause boundary layer considerably surpasses that in the cusp, as evident in Supplementary Fig. 13d and Supplementary Fig. 16d. Moreover, the high-energy electron spectrum in the magnetopause boundary layer is significantly stronger than in the cusp, as seen in Supplementary Fig. 13c and Supplementary Fig. 16c. In Supplementary Fig. 13, the magnetosheath appears to trail the second boundary layer at the magnetopause, identifiable by a more perturbed magnetic field. The ion energy spectrum in the magnetopause boundary layer closely mimics the intense distribution observed in the magnetosheath (Supplementary Fig. 13e, f, g), significantly surpassing the ion flux intensity found in the cusp (Supplementary Fig. 13e, f, g). Most notably, even though the magnetic field within the magnetopause boundary layer is less turbulent compared to the magnetosheath (Supplementary Fig. 13a, b, as indicated by the blue arrow), it remains distinctly different from

the quieter magnetic fields observed in the cusp (Supplementary Fig. 13a, b). These pronounced differences likely arise from the magnetopause boundary layer's closer proximity to the magnetosheath, thereby inheriting certain characteristics.

(2) **The comparison between PSBL at low latitude (Supplementary Fig. 14) and cusp (Supplementary Fig. 16).**

Both the cusp and PSBL at low latitude exhibit similar ion energy spectrum features, which both display protons of ~1 count/s, intermediate ions of ~0.4 count/s, and basically no heavy ions. Nonetheless, the energy level of the enhanced electron spectrum in the PSBL is markedly higher than that observed in the cusp, as delineated in Supplementary Fig. 14d and Supplementary Fig. 16d. In the cusp, the electron energy spectrum is prominently augmented below 100 eV. In contrast, the energy spectrum enhancement in the PSBL is above 100 eV. The expected enhancement of the cusp's energy spectrum is delineated by the red box in Supplementary Fig. 14d, signaling a distinct difference between the two regions. Furthermore, owing to the proximity of the PSBL to the plasma sheet itself, the magnetic field experiences significant perturbations and reductions when the spacecraft becomes close to the plasma sheet (Supplementary Fig. 14b). This is in stark contrast to the relatively stable magnetic fields observed during cusp events (Supplementary Fig. 16b), in which quiet magnetic fields are seen both within the magnetosphere and the cusp region.

(3) **The comparison between PSBL at high latitude (Supplementary Fig. 15) and cusp (Supplementary Fig. 16).**

Analogous to point (2), both the cusp and PSBL at high latitudes display comparable characteristics in their ion energy spectrum. Although the PSBL and the cusp are similar in terms of latitude position, the PSBL demonstrates an electron energy spectrum enhancement markedly exceeding 100 eV as shown in Supplementary Fig. 15d. This is significantly higher than the typical

enhancement ranges observed in the cusp (see Supplementary Fig. 16d), which are 100 eV and below. The expected enhancement of the cusp's energy spectrum is delineated by the red box in Supplementary Fig. 15d, showing a clear difference in the distribution of the electron energy spectrum in the two regions. Moreover, as indicated by the blue arrows in Supplementary Fig. 15b, the magnetic field experiences substantial diminution in regions adjacent to the PSBL, attributed to the spacecraft's closeness to the plasma sheet. This behavior contrasts with the relatively stable magnetic fields observed in the cusp.

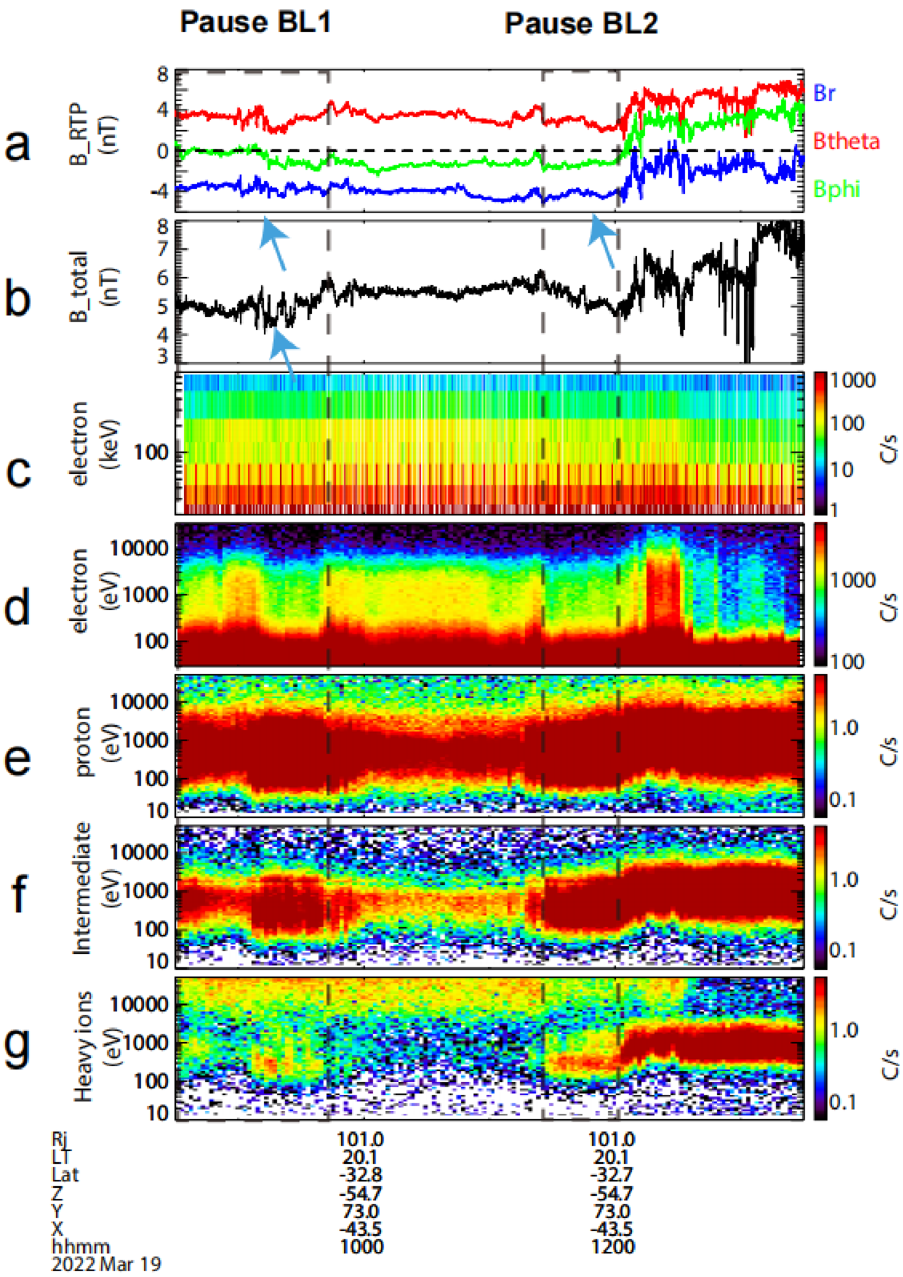

Supplementary Fig. 13. **A magnetopause boundary layer case.** (a) R-Theta-Phi magnetic field components in JSS coordinate; (b) The total magnetic field; (c) The high energy electron spectrogram; (d) The low energy electron spectrogram; Ion Energy spectrogram for protons (e), intermediate ions (f), and heavy ions (g). The black dashed boxes marked the boundary layer regions. The blue arrow points to the disturbed magnetic field.

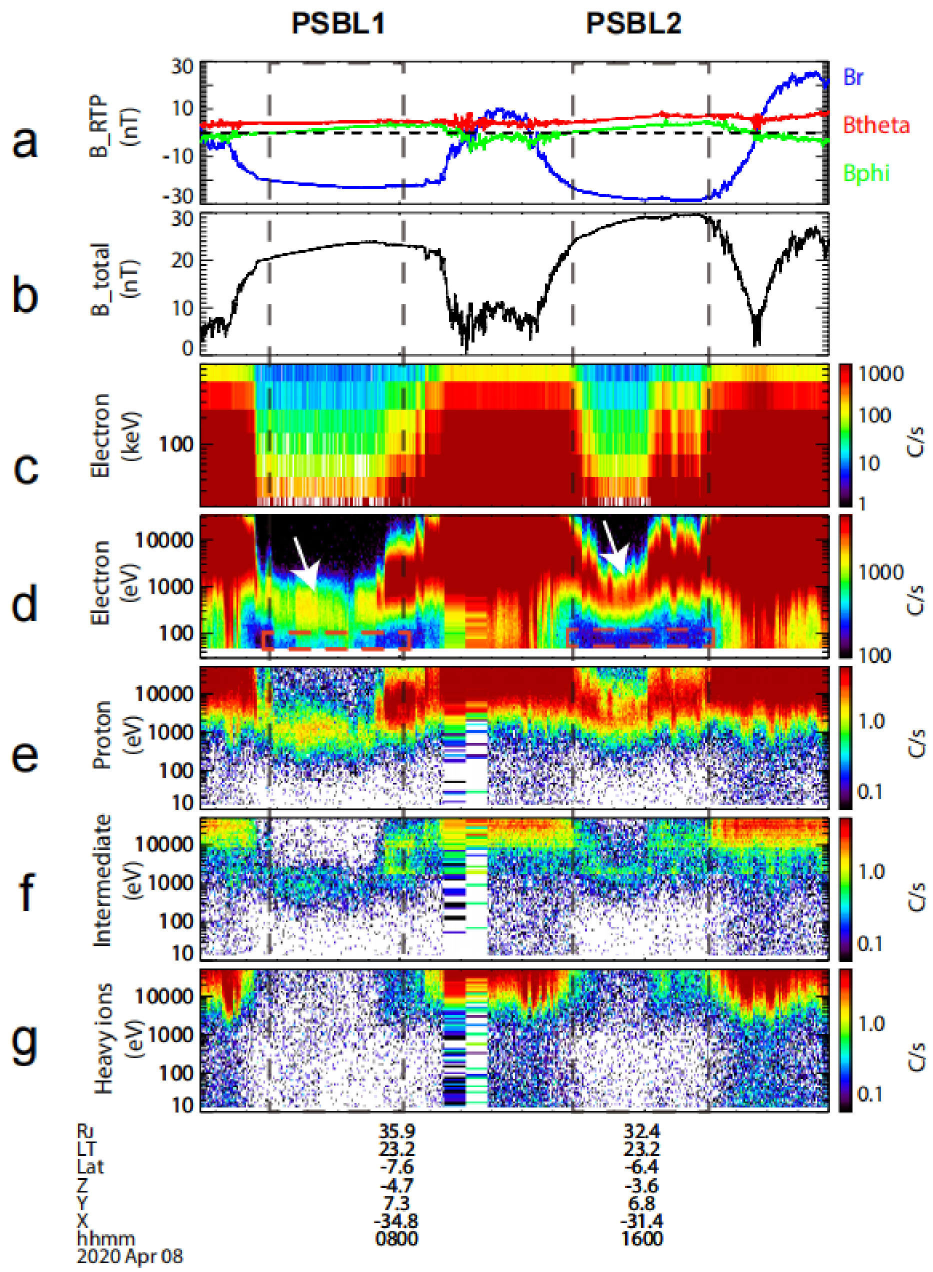

Supplementary Fig. 14. **A plasma sheet boundary layer case at low latitude.** Panel a-g are arranged in the same format as Supplementary Fig. 13. The black dashed boxes marked the boundary layer regions. The white arrow points to the disturbed magnetic field. White arrows point to electron energy spectrum features whose energy is higher than those in the cusp region. The red dashed box indicates the expected electron distribution if it were cusp.

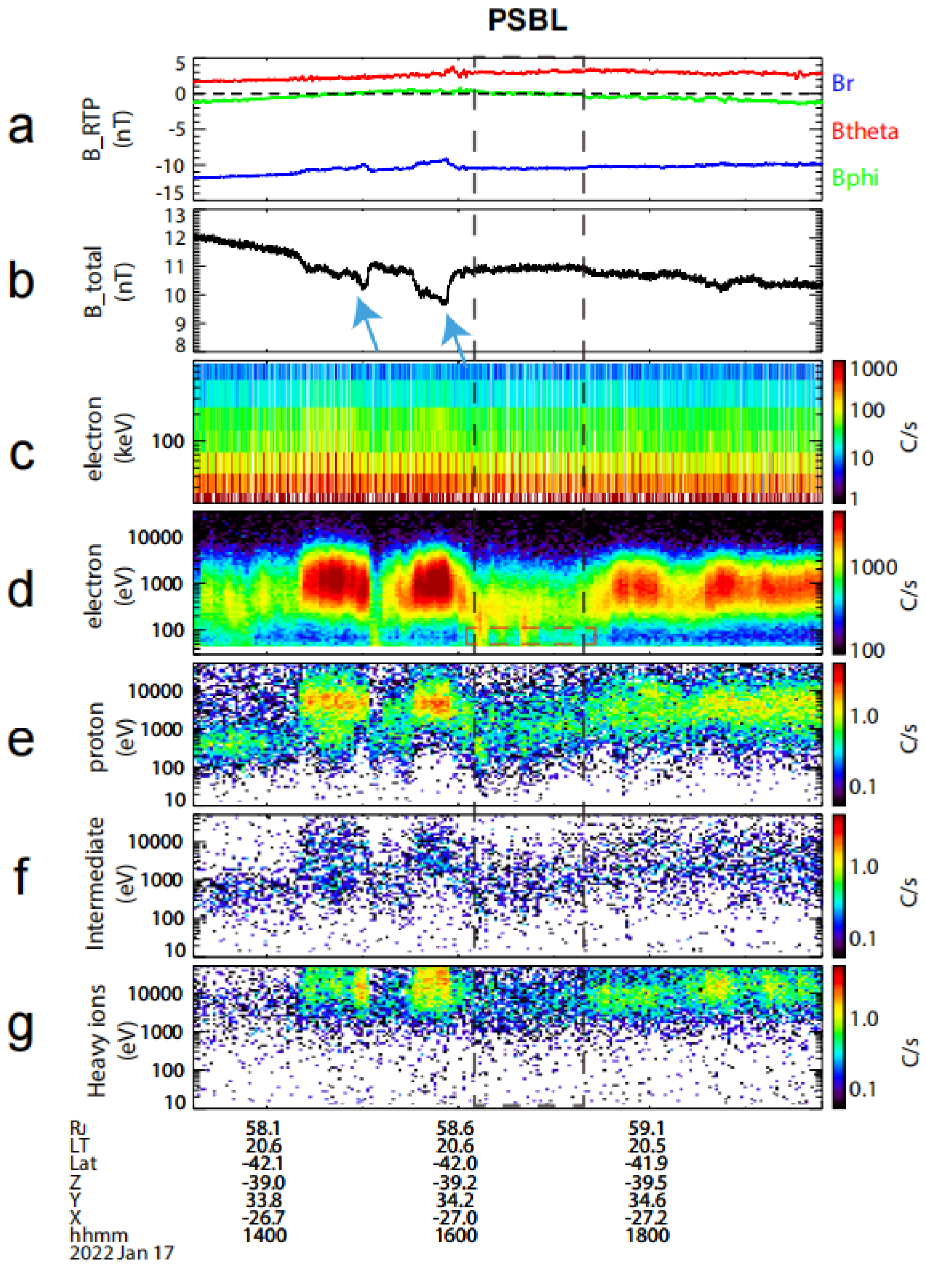

Supplementary Fig. 15. **A plasma sheet boundary layer case at high latitude.** Panel a-g are arranged in the same format as Supplementary Fig. 13. The black dashed boxes marked the boundary layer regions. The blue arrow points to the necessary decrease when the spacecraft is close/enters tha plasma sheet near the PSBL. The red dashed box indicates the expected electron distribution if it were cusp.

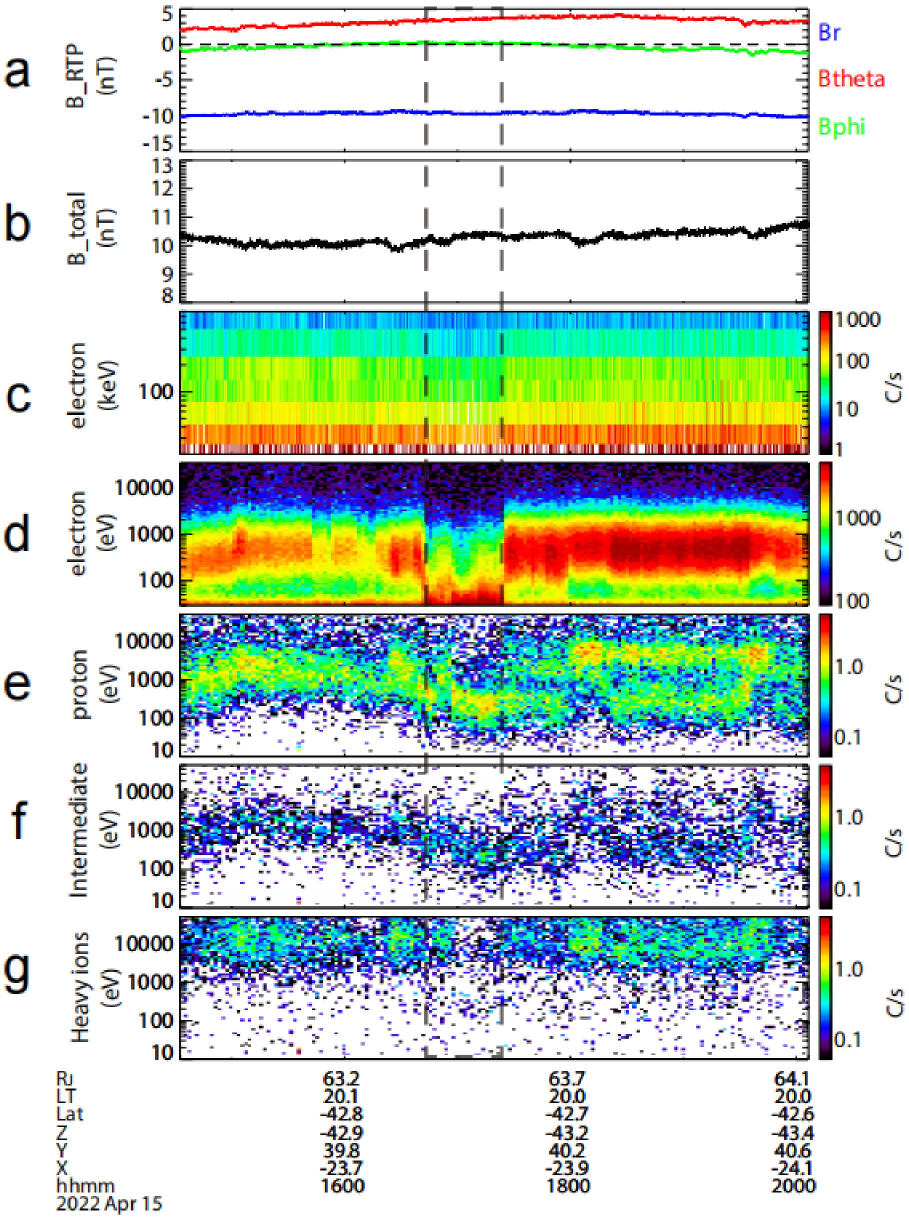

Supplementary Fig. 16. **The cusp case example 6 in this study.** Panel a-g are arranged in the same format as Supplementary Fig. 13. The black dashed boxes marked the cusp regions.

Supplementary References

1. Cargill, P. J. *et al.* Cluster at the Magnetospheric Cusps. *Space Sci Rev* **118**, 321–366 (2005).

2. Spreiter, J. R., Summers, A. L. & Alksne, A. Y. Hydromagnetic flow around the magnetosphere. *Planetary and Space Science* **14**, 223–253 (1966).
3. Smith, M. F. & Lockwood, M. Earth's magnetospheric cusps. *Reviews of Geophysics* **34**, 233–260 (1996).
4. Lin, C. S., Burch, J. L. & Winningham, J. D. Near-conjugate observations of polar cusp electron precipitation using DE 1 and DE 2. *Journal of Geophysical Research: Space Physics* **91**, 11186–11202 (1986).
5. Pitout, F. & Bogdanova, Y. V. The Polar Cusp Seen by Cluster. *Journal of Geophysical Research: Space Physics* **126**, e2021JA029582 (2021).
6. Heikkila, W. J. & Winningham, J. D. Penetration of magnetosheath plasma to low altitudes through the dayside magnetospheric cusps. *Journal of Geophysical Research (1896-1977)* **76**, 883–891 (1971).
7. Frank, L. A. Plasma in the Earth's polar magnetosphere. *Journal of Geophysical Research (1896-1977)* **76**, 5202–5219 (1971).
8. Lockwood, M. *et al.* Ionospheric signatures of pulsed reconnection at the Earth's magnetopause. *Nature* **361**, 424–428 (1993).
9. Newell, P. T. & Meng, C.-I. The cusp and the cleft/boundary layer: Low-altitude identification and statistical local time variation. *Journal of Geophysical Research: Space Physics* **93**, 14549–14556 (1988).
10. Newell, P. T., Meng, C.-I., Sibeck, D. G. & Lepping, R. Some low-altitude cusp dependencies on the interplanetary magnetic field. *Journal of Geophysical Research: Space Physics* **94**, 8921–8927 (1989).

11. Jasinski, J. M. *et al.* Cusp observation at Saturn's high-latitude magnetosphere by the Cassini spacecraft. *Geophysical Research Letters* **41**, 1382–1388 (2014).
12. Jasinski, J. M. *et al.* Cassini plasma observations of Saturn's magnetospheric cusp. *Journal of Geophysical Research: Space Physics* **121**, 12,047-12,067 (2016).
13. Arridge, C. S. *et al.* Cassini observations of Saturn's southern polar cusp. *Journal of Geophysical Research: Space Physics* **121**, 3006–3030 (2016).
14. Lavraud, B. *et al.* Cluster survey of the high-altitude cusp properties: a three-year statistical study. *Annales Geophysicae* **22**, 3009–3019 (2004).
15. Tsyganenko, N. A. & Russell, C. T. Magnetic signatures of the distant polar cusps: Observations by Polar and quantitative modeling. *Journal of Geophysical Research: Space Physics* **104**, 24939–24955 (1999).
16. Dunlop, M. W. *et al.* Cluster Observations of the CUSP: Magnetic Structure and Dynamics. *Surv Geophys* **26**, 5–55 (2005).
17. Zhang, B. *et al.* Predicting the location of polar cusp in the Lyon-Fedder-Mobarry global magnetosphere simulation. *Journal of Geophysical Research: Space Physics* **118**, 6327–6337 (2013).
18. Zhou, X. W., Russell, C. T., Le, G., Fuselier, S. A. & Scudder, J. D. Factors controlling the diamagnetic pressure in the polar cusp. *Geophysical Research Letters* **28**, 915–918 (2001).
19. Shi, Q. Q. *et al.* Spatial structures of magnetic depression in the Earth's high-altitude cusp: Cluster multipoint observations. *Journal of Geophysical Research: Space Physics* **114**, (2009).

20. Jasinski, J. M. *et al.* Diamagnetic depression observations at Saturn's magnetospheric cusp by the Cassini spacecraft. *Journal of Geophysical Research: Space Physics* **122**, 6283–6303 (2017).
21. Spreiter, J. R., Alksne, A. Y. & Abraham-Shrauner, B. Theoretical proton velocity distributions in the flow around the magnetosphere. *Planetary and Space Science* **14**, 1207–1220 (1966).
22. Winslow, R. M. *et al.* Observations of Mercury's northern cusp region with MESSENGER's Magnetometer. *Geophysical Research Letters* **39**, (2012).
23. Raines, J. M. *et al.* Structure and dynamics of Mercury's magnetospheric cusp: MESSENGER measurements of protons and planetary ions. *Journal of Geophysical Research: Space Physics* **119**, 6587–6602 (2014).
24. Slavin, J. A. *et al.* MESSENGER observations of Mercury's dayside magnetosphere under extreme solar wind conditions. *Journal of Geophysical Research: Space Physics* **119**, 8087–8116 (2014).
25. Poh, G. *et al.* MESSENGER observations of cusp plasma filaments at Mercury. *Journal of Geophysical Research: Space Physics* **121**, 8260–8285 (2016).
26. Reiff, P. H., Hill, T. W. & Burch, J. L. Solar wind plasma injection at the dayside magnetospheric cusp. *Journal of Geophysical Research (1896-1977)* **82**, 479–491 (1977).
27. Maynard, N. C. *et al.* Magnetospheric boundary dynamics: DE 1 and DE 2 observations near the magnetopause and cusp. *Journal of Geophysical Research: Space Physics* **96**, 3505–3522 (1991).

28. Øieroset, M., Sandholt, P. E., Denig, W. F. & Cowley, S. W. H. Northward interplanetary magnetic field cusp aurora and high-latitude magnetopause reconnection. *Journal of Geophysical Research: Space Physics* **102**, 11349–11362 (1997).

29. Bosqued, J. M. *et al.* Evidence for ion energy dispersion in the polar cusp related to a northward-directed IMF. *Advances in Space Research* **5**, 149–153 (1985).

30. Pitout, F., Escoubet, C. P., Klecker, B. & Dandouras, I. Cluster survey of the mid-altitude cusp – Part 2: Large-scale morphology. *Annales Geophysicae* **27**, 1875–1886 (2009).

31. Woch, J. & Lundin, R. Magnetosheath plasma precipitation in the polar cusp and its control by the interplanetary magnetic field. *Journal of Geophysical Research: Space Physics* **97**, 1421–1430 (1992).

32. Lockwood, M. & Smith, M. F. Low and middle altitude cusp particle signatures for general magnetopause reconnection rate variations: 1. Theory. *Journal of Geophysical Research: Space Physics* **99**, 8531–8553 (1994).

33. Haerendel, G., Paschmann, G., Sckopke, N., Rosenbauer, H. & Hedgecock, P. C. The frontside boundary layer of the magnetosphere and the problem of reconnection. *Journal of Geophysical Research: Space Physics* **83**, 3195–3216 (1978).

34. Bosqued, J. M. *et al.* Multipoint observations of transient reconnection signatures in the cusp precipitation: A Cluster-IMAGE detailed case study. *Journal of Geophysical Research: Space Physics* **110**, (2005).

35. Escoubet, C. P. *et al.* Temporal evolution of a staircase ion signature observed by Cluster in the mid-altitude polar cusp. *Geophysical Research Letters* **33**, (2006).

36. Chen, S.-H. *et al.* Exterior and interior polar cusps: Observations from Hawkeye. *Journal of Geophysical Research: Space Physics* **102**, 11335–11347 (1997).

37. Fung, S. F., Eastman, T. E., Boardsen, S. A. & Chen, S.-H. High-altitude cusp positions sampled by the Hawkeye satellite. *Physics and Chemistry of the Earth* **22**, 653–662 (1997).

38. Chen, J. *et al.* Cusp energetic particle events: Implications for a major acceleration region of the magnetosphere. *Journal of Geophysical Research: Space Physics* **103**, 69–78 (1998).

39. Gurnett, D. A. & Frank, L. A. Plasma waves in the polar cusp: Observations from Hawkeye 1. *Journal of Geophysical Research: Space Physics* **83**, 1447–1462 (1978).

40. Maggs, J. E. coherent generation of VLF hiss. *Journal of Geophysical Research (1896-1977)* **81**, 1707–1724 (1976).

41. Gurnett, D. A., Kurth, W. S. & Scarf, F. L. Auroral hiss observed near the Io plasma torus. *Nature* **280**, 767 (1979).

42. Stone, R. G. *et al.* Ulysses radio and plasma wave observations in the jupiter environment. *Science* **257**, 1524–1531 (1992).

43. Nykyri, K., Otto, A., Adamson, E., Kronberg, E. & Daly, P. On the origin of high-energy particles in the cusp diamagnetic cavity. *Journal of Atmospheric and Solar-Terrestrial Physics* **87–88**, 70–81 (2012).

44. Nykyri, K. *et al.* Cluster observations of magnetic field fluctuations in the high-altitude cusp. *Annales Geophysicae* **22**, 2413–2429 (2004).

45. Grison, B. *et al.* Wave particle interactions in the high-altitude polar cusp: a Cluster case study. *Annales Geophysicae* **23**, 3699–3713 (2005).

46. Balogh, A. *et al.* THE CLUSTER MAGNETIC FIELD INVESTIGATION. *Space Science Reviews* **79**, 65–91 (1997).

47. Johnstone, A. D. *et al.* Peace: A Plasma Electron and Current Experiment. in *The Cluster and Phoenix Missions* (eds. Escoubet, C. P., Russell, C. T. & Schmidt, R.) 351–398 (Springer Netherlands, Dordrecht, 1997). doi:10.1007/978-94-011-5666-0_13.

48. Rème, H. *et al.* The Cluster Ion Spectrometry (CIS) Experiment. in *The Cluster and Phoenix Missions* (eds. Escoubet, C. P., Russell, C. T. & Schmidt, R.) 303–350 (Springer Netherlands, Dordrecht, 1997). doi:10.1007/978-94-011-5666-0_12.

49. Décréau, P. M. E. *et al.* WHISPER, A RESONANCE SOUNDER AND WAVE ANALYSER: PERFORMANCES AND PERSPECTIVES FOR THE CLUSTER MISSION. *Space Science Reviews* **79**, 157–193 (1997).

50. Dougherty, M. K. *et al.* The Cassini Magnetic Field Investigation. in *The Cassini-Huygens Mission: Orbiter In Situ Investigations Volume 2* (ed. Russell, C. T.) 331–383 (Springer Netherlands, Dordrecht, 2004). doi:10.1007/978-1-4020-2774-1_4.

51. Young, D. T. *et al.* Cassini Plasma Spectrometer Investigation. *Space Science Reviews* **114**, 1–112 (2004).

52. Chen, J. *et al.* Prediction of Axial Asymmetry in Jovian Magnetopause Reconnection. *Geophysical Research Letters* **50**, e2022GL102577 (2023).

53. Eather, R. H. & Mende, S. B. Airborne observations of auroral precipitation patterns. *Journal of Geophysical Research (1896-1977)* **76**, 1746–1755 (1971).

54. Eather, R. H. Auroral proton precipitation and hydrogen emissions. *Reviews of Geophysics* **5**, 207–285 (1967).

55. Gérard, J.-C. *et al.* Saturn's auroral morphology and activity during quiet magnetospheric conditions. *Journal of Geophysical Research: Space Physics* **111**, (2006).

56. Grodent, D., Gérard, J.-C., Cowley, S. W. H., Bunce, E. J. & Clarke, J. T. Variable morphology of Saturn's southern ultraviolet aurora. *Journal of Geophysical Research: Space Physics* **110**, (2005).

57. Carbary, J. F. The morphology of Saturn's ultraviolet aurora. *Journal of Geophysical Research: Space Physics* **117**, (2012).

58. Clarke, J. T. Auroral Processes on Jupiter and Saturn. in *Geophysical Monograph Series* (eds. Keiling, A., Donovan, E., Bagenal, F. & Karlsson, T.) 113–122 (American Geophysical Union, Washington, D. C., 2013). doi:10.1029/2011GM001199.

59. Greathouse, T. *et al.* Local Time Dependence of Jupiter's Polar Auroral Emissions Observed by Juno UVS. *Journal of Geophysical Research: Planets* **126**, e2021JE006954 (2021).

60. Sulaiman, A. H. *et al.* Jupiter's Low-Altitude Auroral Zones: Fields, Particles, Plasma Waves, and Density Depletions. *Journal of Geophysical Research: Space Physics* **127**, e2022JA030334 (2022).

61. Zhang, B. *et al.* How Jupiter's unusual magnetospheric topology structures its aurora. *Science Advances* **7**, eabd1204 (2021).

62. Ebert, R. W., Bagenal, F., McComas, D. J. & Fowler, C. M. A survey of solar wind conditions at 5 AU: a tool for interpreting solar wind-magnetosphere interactions at Jupiter. *Front. Astron. Space Sci.* **1**, (2014).

63. Nichols, J. D., Cowley, S. W. H. & McComas, D. J. Magnetopause reconnection rate estimates for Jupiter's magnetosphere based on interplanetary measurements at ~5AU. *Annales Geophysicae* **24**, 393–406 (2006).

64. Nichols, J. D. *et al.* Response of Jupiter's auroras to conditions in the interplanetary medium as measured by the Hubble Space Telescope and Juno. *Geophysical Research Letters* **44**, 7643–7652 (2017).

65. Zhang, X.-J. *et al.* Plasma Sheet Boundary Layer in Jupiter's Magnetodisk as Observed by Juno. *Journal of Geophysical Research: Space Physics* **125**, e2020JA027957 (2020).
